# Rabies surveillance and prevention in Guinea: Epidemiological data and postexposure prophylaxis challenges

**DOI:** 10.14202/vetworld.2024.1828-1835

**Published:** 2024-08-20

**Authors:** Aissatou Touré, Madi Savadogo, Mohamed Idriss Doumbouya, Fassou Kourouma, Pépé Gbamou, Zékiba Tarnagda, Rianatou Bada-Alambedji

**Affiliations:** 1 National Directorate for Veterinary Services, Ministry of Agriculture and Livestock, P.O Box 576, Conakry, Guinea; 2 Department of Public Health and Environment, Ecole Inter-State School of Veterinary Sciences and Medicine, P.O Box 5077, Dakar, Senegal; 3 National Influenza Reference Laboratory, Unit of Epidemic potential Diseases, Emerging Diseases and Zoonoses, Department of Medical Biology and Public Health Research Institute for Health Sciences (IRSS/CNRST), P.O. Box 7047, Ouagadougou, Burkina Faso; 4 Fundamental and Applied Research for Animals and Health, Faculty of Veterinary Medicine, University of Liege, Quartier Vallée 2 avenue de Cureghem, 6, Liege, Belgium; 5 Directorate of Animal Health, Directorate General of Veterinary Services, Ministry of Agriculture, Animal et Halieutic Resources, P.O Box 7026, Ouagadougou, Burkina Faso

**Keywords:** animal bite management, cats, cattle, dogs, fluorescent antibody test, Guinea, humans, monkeys, rabies

## Abstract

**Background and Aim:**

Canine rabies is an endemic form of zoonosis and represents a major public health threat in Guinea, similar to other African countries. However, few investigations on the epidemiology of rabies in animals and humans have been conducted, and evidence-based data required to inform health policies remain inadequate. This study was conducted to update our knowledge of human dog-mediated rabies epidemiology and post-exposure prophylaxis (PEP) accessibility-related factors in Guinea.

**Materials and Methods:**

This retrospective study, conducted from January 2018 to December 2020, collected data on animal bite cases, veterinary observations, rabies diagnoses through fluorescent antibody test, and PEP delivery from three veterinary and medical entities. Statistical analysis utilized Chi-square test and Fisher’s exact test to evaluate relationships between variables.

**Results:**

An average of 775 bites was recorded annually, and dogs were responsible for 98% of bites. However, only 64% of the biting dogs were under veterinary observation as required for integrated bite case management. Regarding the geographical distribution of bite cases, the entire country was affected, with the highest number of bites recorded in the prefectures of Nzérékoré and the special zone of Conakry. In addition, the laboratory diagnosis of brain samples from biting dogs indicated that 72% of the samples were rabies-positive. However, regarding prevention, only 58% of the bitten individuals received full PEP.

**Conclusion:**

Improving disease surveillance and PEP provision for dog-transmitted rabies is crucial to preventing human cases and deaths. Increasing community awareness is essential for enhancing dog vaccination and PEP utilization. A national action plan integrating stakeholders for controlling canine rabies should be developed for effective One Health collaboration.

## Introduction

Rabies, which can be fatal for humans and various domesticated and wild animals, is an infectious disease. Each year, rabies claims over 59,000 human lives across more than 150 nations [1]. According to the World Health Organization (WHO) Rabies Modeling Consortium’s estimation based on the current epidemiological situation, there could be over one million fatalities due to rabies in 67 African and Asian countries between 2020 and 2035 [2]. About 100% canine rabies prevention can be accomplished through timely pre-exposure or post-exposure prophylaxis (PEP) for high-risk communities and a sustained vaccination coverage of at least 70% in the dog population. To increase the commitment of the international community and stakeholders in the control of human dog-mediated rabies, the global strategy regarding the elimination of human dog-mediated rabies by 2030 recommends One Health collaboration at national, regional, and international levels [2, 3]. For example, by implementing an integrated bite case management (IBCM) approach in Tanzania, Lushasi *et al*. [3] demonstrated the effectiveness of multisectoral collaboration in improving rabies case detection, intersectoral communication, PEP provision for bite victims, and follow-up of cases. It is expected that in line with the 2030 global target of a world safe from rabies, each endemic country will develop an integrated national strategic plan for coordinated and sustained national rabies control efforts. Based on the disease transmission cycle, nearly 99% of human cases are caused by infected dogs in African countries, as reported by Ribadeau-Dumas *et al*. [4]. Therefore, dog rabies vaccination represents a meaningful approach to be included in rabies elimination strategic plans, as well as surveillance, PEP, raising awareness, building diagnostic capacity, and policy development.

Guinea, a low-resource West African country, is endemic to human dog-mediated rabies [5, 6]. In sub-regional countries, local communities use dogs for various socio-economic functions, such as house guarding, herd protection, hunting, and companionship, particularly for children [7, 8]. In Guinea, these community practices have led to a growing number of dogs. Dog ownership often involves free roaming and inadequate vaccination coverage [5, 8]. For example, a vaccination coverage rate of 3% for biting dogs was reported in the country between 2002 and 2012 [5]. Frequent dog bites are reported due to their open access to communities, primarily affecting children [9]. There are several limitations, including limited knowledge, lack of integration, vaccine shortages, and financial and geographical barriers, to the management of bite cases and the provision of PEP. Overall, the disease remains poorly monitored and under-reported in both animals and humans. In many cases, limited data collected by entities involved in rabies control are rarely analyzed and disseminated to relevant stakeholders, including policymakers. To strengthen control efforts, rabies was selected as one of the five priority zoonotic diseases for multi-sectoral engagement in the country [6]. The epidemiology of rabies in Guinea remains uncertain. A previous study on rabies in the country has focused solely on Conakry city [5]. This study aimed to gather data about rabies epidemiology and control in 33 provinces and Conakry’s special administrative area.

## Materials and Methods

### Ethical approval

This study did not require ethical approval. This study was conducted with the collaboration and technical support of national agencies in charge of animal and human rabies prevention and control. All data were collected from anonymized databases provided by national competent authorities: The Directorate of National Veterinary Services and the Diagnosis and Veterinary Central Laboratory of the Ministry of Livestock, and the National Health Security Agency and health centers of the Ministry of Health.

### Study period and location

**Figure-1:**
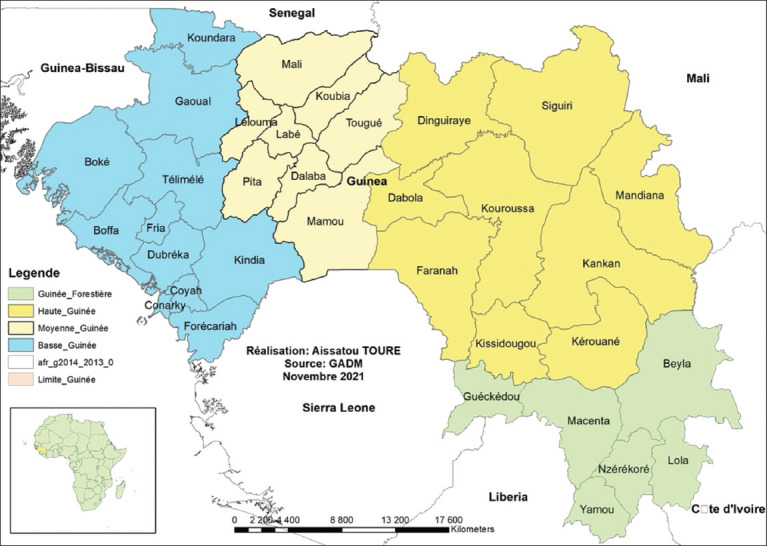
Geographical situation of the 33 prefectures and the special administrative area of Conakry covered by this study (Source: https://gadm.org/maps/GIN_1.html).

The study consisted of a retrospective survey of data collected between January 2018 and December 2020 in Guinea. The survey covered the special administrative area of Conakry and the 32 prefectures of the country, as presented in Figure-1: six located in the administrative region of Forestry Guinea (Guinée Forestière), eight in the administrative region of Middle Guinea (Moyenne Guinée), eight in the administrative region in Maritime Guinea (Guinée Maritime or Basse Guinée), and 10 in the administrative region of Haute Guinea (Haute Guinée), including the special area of the city of Conakry. Guinea shares borders with multiple rabies-endemic countries such as Cote d’Ivoire [10], Liberia [11, 12], Guinea-Bissau [13], Mali [14], Senegal [15], and Sierra Leone [16].

### Data collection

The survey focused on data related to rabies in animals and humans. Data were obtained from databases provided by technical entities involved in the management of rabies in Guinea. These entities included the National Directorate of Veterinary Services Direction Nationale des Services Vétérinaires in charge of animal rabies prevention and control (e.g. surveillance of animal rabies, dog vaccination, observation of biting dogs based on a 15-day duration), Veterinary and Diagnosis Central Laboratory (Laboratoire Central Vétérinaire et de Diagnostic, [LCVD]) in charge of rabies diagnosis using the fluorescent antibody test (FAT) (samples collection, laboratory analysis), and National Agency for Health Security Agence Nationale de Sécurité Sanitaire in charge of human rabies prevention (pre-exposure prophylaxis, PEP using the 4-dose Zagreb regimen, surveillance, and data collection).

For the survey, guides were developed to register data collected from the databases by the authors. For each study site, variables of interest related to biting dogs (sex, number, species, management, and vaccination status), animal samples tested using the FAT (sex, age, sample quality, and results of diagnosis), and bite victims (sex, age, professional status, vaccination status of biting dogs, PEP provision status, and follow-up of outcome for the bite victims) were considered.

### Statistical analysis

All obtained data were recorded in Microsoft Excel 2016 (Microsoft Office, Washington, USA) and transferred to Rx3.6.3 for data analysis. Descriptive statistics (proportions and means), Chi-square test, and Fischer’s exact test were computed to assess the association between variables (rabies diagnosis results, completeness of PEP) and explanatory variables (characteristics of dogs, locations, quality of samples, and characteristics of bitten persons). A 95% confidence level was considered, and statistical significance was set at p < 0.05.

## Results

### Situation of bites caused by suspected rabies

**Figure-2:**
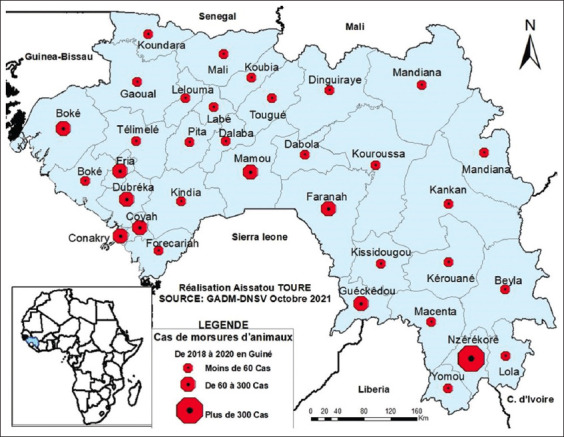
Geographical distribution of bite cases in Guinea from 2018 to 2020 (Source: https://gadm.org/maps/GIN_1.html).

During the study period, 2,326 biting animals were reported by veterinary services throughout the country, corresponding to an average of 775 animals registered per year. Up to 42.27% of biting animals were registered in 2020 (Table-1). Of all reported biting animals, 97.81% were dogs. Overall, 88.61% of bite victims were humans and 9.29% were production animals. Concerning the management of biting animals, 1490 biting animals (64.06%) were put under veterinary observation as required by national procedures: Owned dogs (97.85%), cats (1.48%), and monkeys (0.67%). Figure-2 presents the geographical distribution of reported biting animals throughout the study country. Higher numbers of biting animals were reported in the prefecture of Nzérékoré (30.66%) and the special area of Conakry City (15.09%). However, only one biting animal was reported in Mali’s prefecture between January 2018 and December 2020.

**Table-1:** Data on bites caused by suspected rabies in Guinea by 2018–2020.

Variables	Number of bites	Percentage
Year of occurrence		
2018	583	25.06
2019	760	32.67
2020	983	42.27
Species of biting animals		
Dog	2275	97.81
Cat	37	1.59
Monkey	14	0.60
Management of biting animals		
Under veterinary observation	1490	64.06
No veterinary observations	836	35.94
Category of bitten victims		
Human	2061	88.61
Dog	49	2.10
Cattle	216	9.29
Total	2326	100

### Laboratory diagnosis of rabies in biting animals

Regarding the laboratory confirmation of rabies in biting dogs, only 47 brain samples were received from the Veterinary and Diagnosis Central Laboratory from January 2018 to December 2020. Samples were collected from eight prefectures (Table-2). Of all samples subjected to the FAT, 72.34% were positive for the rabies virus. The number of positive cases was higher in reported unowned dogs (80.95%) than in owned dogs. In addition, male dogs and dogs younger than 4 years were the most represented in the positive samples compared with female dogs and dogs aged 4 years and older. However, no significant association was observed between the virological status of biting dogs and characteristics such as age, sex, quality of samples, and ownership (owned or un-owned) (p > 0.05, Table-2).

**Table-2:** Association between locations, sample quality, characteristics of dogs, and samples’ virological status.

Variable	Number of samples tested	Number of positive samples	% of positive samples (95% CI)	p-value
Ownership status of dogs				
Un-owned	21	17	80.95 (64.1–97.7)	0.53
Owned	26	17	65.38 (46.2–80.6)	
Sex of dogs				
Male	29	23	79.31 (61.6–90.2)	0.17
Female	16	9	56.25 (33.2–76.9)	
Unkown	2	2	100 (NA)	
Age of dogs (years)				
4	30	19	63.33 (45.5–78.1)	0.15
From 4 to 6	7	6	85.71 (48.7–97.4)	
Unkown	10	9	90.00 (59.6–98.2)	
Locations				
Conakry	5	1	20.00 (1.2–29.8)	NA
Kindia	1	1	100 (NA)	
Boké	10	3	30 (10.8–60.3)	
Mamou	2	2	100 (NA)	
Labé	5	4	80.00 (37.6–96.4)	
Faranah	17	12	70.59 (46.9–86.7)	
Kankan	4	4	100 (NA)	
Nzérékoré	3	3	100 (NA)	
Year of sampling				
2018	7	4	57.14 (18.4–90.1)	NA
2019	27	20	74.07 (53.7–88.9)	
2020	13	10	76.92 (46.2–94.9)	
Quality of the samples				
Good	17	5	70.59 (44.0–89.6)	0.51
Acceptable	25	7	76.00 (54.9–90.6)	
Putrefied	5	2	60.00 (14.6–94.7)	

### Complete PEP in bite victims

During the 3-year study period, 3942 bite victims were registered with the National Agency for Health Security. All PEPs were provided based on a 4-dose Zagreb regimen during the study period (Table-3). Overall, 58.09% of bite victims received a full PEP (four doses of rabies vaccine as follows: Two doses on day 0, one dose on day 7, and one dose on day 21). The average age of the exposed persons was significantly different between males (33.84) and females (31.89, p < 0.05). The completeness of PEP was variable according to the vaccination status of the biting dog, ownership status of the biting dog, age of the bite victims, professional status, and year of bite occurrence. However, bite victims of males were significantly associated with the PEP completeness status, with male bite victims being more likely to receive a complete PEP (p < 0.05).

**Table-3:** Association between completeness of PEP and biting animal characteristics, bitten persons, and year of bite occurrence.

Variables	Completeness of post-exposure prophylaxis received (based on the 4-dose Zagreb regimen)				p-value

	Day 21: Fourth dose provided (full PEP) (%)	Day 7: Third dose provided (%)	Day 0: Two doses provided (%)	No PEP provided (%)	
Vaccination status of biting dogs					
Vaccinated	11.57	1.70	1.24	8.37	0.25
Non-vaccinated	30.24	11.22	2.64	12.00	
Unknown	16.29	1.80	0.00	2.94	
Ownership status of biting dogs					
Owned dog	2.96	2.11	0.46	2.64	0.86
Unknown dog	44.22	8.98	2.58	15.15	
Killed dog (unknown)	10.91	3.62	0.84	5.53	
Sex of bite victims					
Male	39.50	3.93	2.36	9.97	0.01
Female	18.59	10.78	1.52	13.35
Age of bite victims (in years)					
<10	17.48	4.16	1.32	8.04	0.99
10–20	15.02	4.36	1.22	7.51	
21–30	6.37	1.93	0.33	2.61	
31–40	8.32	2.56	0.38	2.66	
41–50	4.44	0.66	0.28	1.45	
51 and over	6.47	1.04	0.36	1.04	
Professional status of bite victims					
Schoolchild	17.68	4.26	2.36	7.28	0.97
Employee	8.98	1.75	0.30	2.59	
Self-employed	6.11	1.24	0.23	1.34	
Unemployed	14.23	2.69	0.48	8.07	
Housewife	6.47	3.15	0.33	3.09	
Unknown	4.62	1.62	0.18	0.94	
Year of bite occurrence					
2018	15.78	1.80	0.86	8.95	0.52
2019	29.78	7.08	2.56	10.58	
2020	12.53	5.83	0.46	3.78	

PEP=Post-exposure prophylaxis

## Discussion

Effective rabies control calls for an integrated approach combining animal and human health policies informed by epidemiological data and socioeconomic factors. Thus, appropriate surveillance allows the establishment of evidence-based data on the burden to inform policy and resource mobilization for the fight against rabies [17]. In Guinea, this study reveals that suspected rabies bites impact both humans and production animals. These observations are consistent with conclusions from previous studies by Hampson *et al*. [1], Keita *et al*. [14], and Beyene *et al*. [18] on the high impact of rabies on public and animal health, leading to significant economic losses associated with a decline in the living conditions of vulnerable communities. In addition, this highlights the need to increase awareness raising on the role that farming animals (e.g., goats) can play in transmitting rabies to humans [19]. Therefore, the elimination of this disease can contribute to improving the socioeconomic conditions of populations, including their ability to access health facilities and care. In addition, almost all reported bite cases were caused by dogs, highlighting the role of this animal species in the spread of the disease in animal populations and its transmission to humans. In fact, the previous investigations by Youla *et al*. [5], Traoré *et al*. [20], and Savadogo *et al*. [21] conducted in Guinea, Mali, and Burkina Faso revealed that dogs were the main animal species responsible for the transmission of human rabies. Therefore, dog vaccination remains the best pathway to break the epidemiological cycle of rabies and stop its transmission to humans. In Chad, Zinsstag *et al*. [22] demonstrated that vaccination coverage of 70% after sustained annual vaccination interrupts the rabies virus circulation among dogs and is thus more cost-effective than PEP alone for preventing human rabies. Moreover, another significant limitation of PEP management is the absence of information regarding the different categories of recorded bite wounds. However, the PEP must be provided to bitten persons based on categories (I, II, and III) defined by the WHO Strategic Advisory Group of Experts on rabies immunization [23].

Veterinary consultation and rabies observation are crucial when dealing with potentially rabid biting dogs for surveillance and PEP. In assessing the risk of infection in bitten animals, veterinarians use clinical observation as a method. Its application ensures sufficient PEP for exposed individuals while reducing the need for rabies vaccines and immunoglobulins. The study found that a significant number of biting dogs had not previously been examined by a vet. This situation could be explained by a lack of community awareness, low access of dog owners to veterinary services, and a lack of adequate logistics for the secured veterinary observation of biting dogs [24, 25]. In addition, the limited use of veterinary observation of biting dogs reveals the urgent need for an operational IBCM system to improve One-Health collaboration between medical and veterinary services [3].

The rabies virus was detected at high levels in the canine population based on laboratory findings. The high prevalence of rabies in infected dogs underscores the high risk of transmission to the population. The reported number of annual samples investigated in Guinea is among the lowest in the sub-region [26–28], suggesting under-reporting of the disease. This failure of the existing surveillance systems to capture the real burden in animals and humans may explain the low political will for rabies control, unlike other diseases such as malaria, Ebola virus, and coronavirus disease 2019 (COVID-19). Neglecting rabies increases the lack of awareness (in professionals and community members) and evidence-based data on the associated burden, reinforcing its neglect during national public health and animal health policy design [17, 29]. Finally, the result is low political engagement, which Dodet [30] described as a “vicious circle of indifference” about rabies.

The findings also showed that a significant proportion of bite victims did not receive full PEP. However, rabies is a 100% fatal disease. Fortunately, whether bite victims are timely provided with appropriate PEP, infection by the virus is 100% preventable. Studies in Burkina Faso [9] and Cote d’Ivoire [25] reported similar poor PEP coverage in high-risk individuals. In most cases, the main reasons may include a lack of awareness of rabies prevention in the communities and low geographical and financial accessibility to PEP. Because rabies is a neglected disease, resources are inadequate for its control, and countries are regularly affected by PEP shortages. Thus, this may explain why the proportion of provided PEP was lowest in 2020. Indeed, this period corresponds to the outbreak of the COVID-19 pandemic, during which consequences on rabies control have been observed in several Latin American, Asian, and African countries [31, 32]. Regarding vaccine shortages and financial inaccessibility, it is necessary to envision the introduction of cost-effective PEP regimens. Indeed, it is now known that current WHO pre-qualified human vaccines, when administered via the intra-dermal route instead of the intra-muscular route, the required PEP dose can be divided by 5–10, ensuring an equivalent or higher immune response [23]. Therefore, the adoption of such regimens could reduce the direct costs of vaccines and increase the number of people who can benefit from pre-exposure vaccination as well as the PEP. Overall, most bitten persons were children, probably because they used to play with community dogs outside their homes and on the streets. The predominance of childhood cases among rabies-related deaths was previously reported [1, 9]. Therefore, rabies prevention strategies should include education for school children about vector animals, modes of transmission, and the need to inform parents in case of bite scratch or licking by unknown or community dogs. Such strategies help improve children’s literacy on rabies, including their attitudes toward roaming dogs and actions to take when a dog bite occurs [33]. Overall, the significant discrepancies observed between the number of bite cases, the number of biting dogs put under veterinary observation, the number of samples tested by laboratory, and the number of bitten persons who received PEP to reflect the lack of communication and coordination between entities involved in rabies prevention and control, especially the veterinary and medical sectors. This conclusion is in accordance with the findings reported in Chad [34] and Burkina Faso [35]. To address this gap, the development of a national rabies control strategy should consider IBCM, which has been shown to be effective in other African countries [3]. Effective multi-sectoral collaboration requires all involved stakeholders to be trained on One Health core competencies and understand the added value of improved synergy between relevant disciplines, sectors, and actors.

## Conclusion

This study showed that the rabies virus is circulating in animals in Guinea, with dog bites being the main cause of transmission to humans. The proportion of samples positive for dogs sent for laboratory diagnosis was high. Therefore, there was a high risk of human infection due to low PEP coverage in bitten individuals. In addition, the results revealed a discrepancy between the databases provided by different entities, reflecting a lack of close collaboration between veterinary and medical services in charge of rabies control and prevention.

## Recommendations

The findings highlight key actions for improving the fight against human dog-transmitted rabies in Guinea:

- To effectively raise awareness, a communication plan focusing on dog vaccination, rabies PEP, and the national rabies burden is necessary. Utilize mobile technologies and social media in the awareness strategy.
- Involved medical and veterinary facilities should use standardized data collection tools for effective data collection. Technical staff need comprehensive training covering bite case management, sample collection, shipping, epidemiological data collection, analysis, and reporting.
- In accordance with the global target of ending human dog-transmitted rabies by 2030, the government should create a comprehensive, intersectoral strategic plan for its implementation.

## Authors’ Contributions

AT, MS, ZT, and RBA: Designed, conceptualized, and supervised the study implementation, conducted the study, collected and analyzed the data, and wrote the manuscript. AT: Collected and interpreted data. AT, MS, and MID: Provided research material and analyzed data. MID, FK, PG, and RBA: Interpreted data and logistic support. All authors have read, reviewed, and approved the final manuscript.
